# Experimental study on flow characteristics of jet ventilation in crossflow in confined mine spaces

**DOI:** 10.1038/s41598-024-58267-5

**Published:** 2024-04-05

**Authors:** Jue Wang, Cheng Jiang, Xihua Zhou, Jiayu Kang, Shixuan Yu, Gang Bai

**Affiliations:** 1 School of Civil Engineering, Liaoning Technical University, Liaoning, China; 2 School of Safety Science and Engineering, Liaoning Technical University, Liaoning, China; 3 Key Laboratory of Mine Thermodynamic Disasters and Control of Ministry of Education, Liaoning Technical University, Liaoning, China

**Keywords:** Energy science and technology, Engineering

## Abstract

The increasing depth of mine excavation presents greater challenges in mine ventilation and in managing cooling energy consumption. Therefore, there is an urgent need for comprehensive research on jet ventilation influenced by low-speed crossflows. This study investigated the impact of flow velocity ratios (*R*) and jet exit diameters (*d*) on flow-field distribution and flow characteristics through velocity measurements and smoke flow visualization experiments. The results of the study revealed two distinct types of air lakes formed by jet ventilation in crossflow (JVIC), with one being wall-attached and the other suspended. Notably, a significant secondary flow phenomenon was observed in the near-field near the upper wall. Additionally, the deflection angle (*θ*_j_) of JVIC decreases as *R* and *d/D* increase, leading to the formation and movement of a semi-confined point (SP) and a confined point (CP) in the -*x* direction. Moreover, the wall confinement effect diminishes the jet’s diffusion and deflection ability in the -*z* direction, leading to increased penetration in the *x* direction. Before the formation of the SP, the deflection section of the jet lengthens, followed by a rapid shortening upon its formation. Finally, the study further developed empirical equations for the jet axial trajectory and diffusion width.

## Introduction

**Figure 1:**
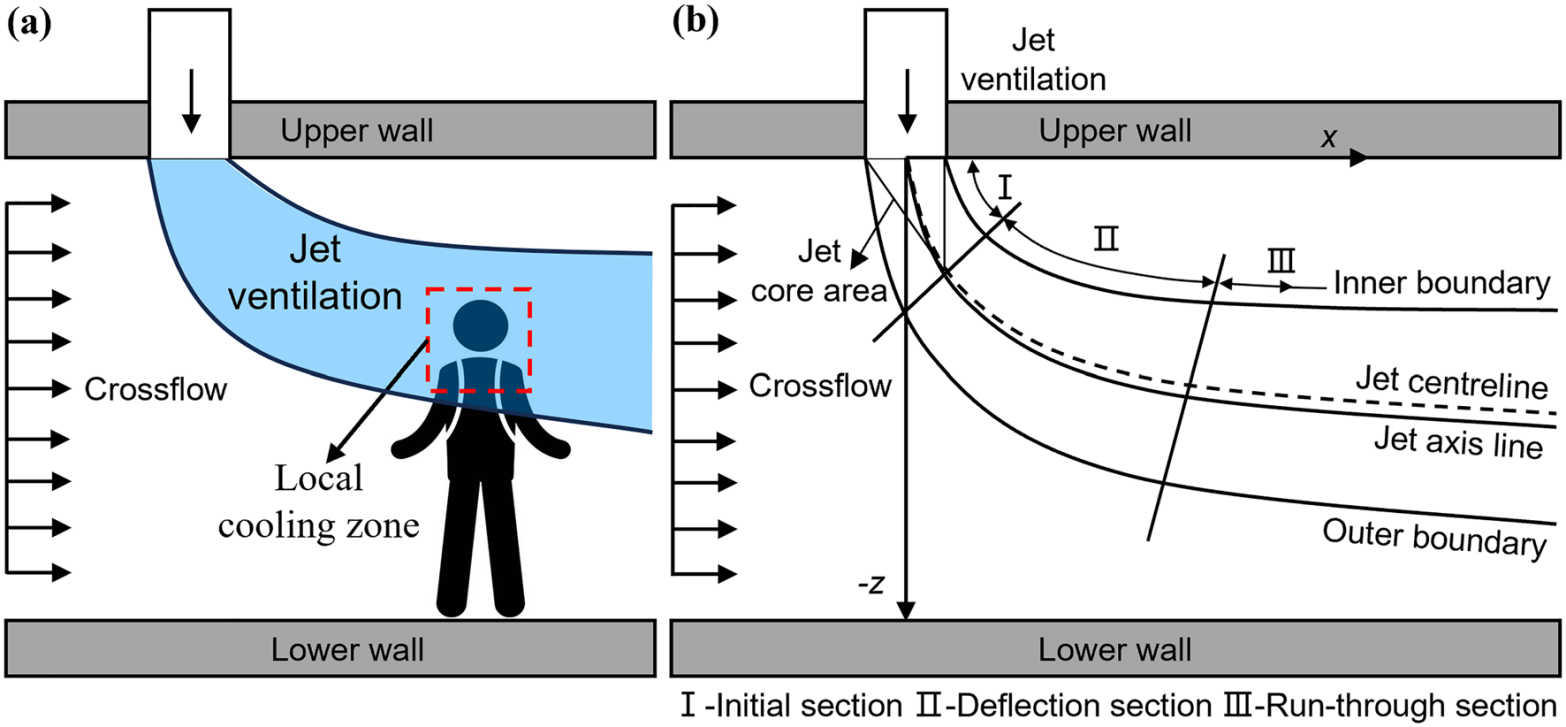
Schematic of the jet ventilation in crossflow: (**a**) local cooling mode, (**b**) theoretical flow-field structure.

In the context of mining operations extending deeper underground, workers are increasingly exposed to occupational hazards due to the elevated temperatures in the environment^1^. The high-temperature airflow present in mines poses a significant threat to workers’ well-being, resulting in a rise in heat-related incidents, consequently impacting operational efficiency^2^. Therefore, effective management of the non-uniform thermal environment in mines is pivotal for preserving the occupational health and safety of workers^3,4^. As a result, the implementation of air conditioning systems for efficient mine ventilation has become increasingly crucial^5^. Additionally, the challenges associated with mine ventilation have been amplified by the escalating depth of mining. The conventional full air cooling mode has been progressively losing efficacy, leading to issues of non-uniform air supply and increasing energy consumption. Against the backdrop of the ongoing energy transition and the control requirements of the non-uniform thermal environment, a comprehensive investigation into the efficacy of localized jet ventilation becomes of paramount importance to enhance the comfort of mine workers' working environment and promote sustainable development. Therefore, based on the actual mine space characteristics and personnel wearing characteristics underground, the team proposed a head-neck local ventilation cooling mode for mine workers as shown in Fig. 1a. The mode designates workers' head-neck as an efficient cooling zone^6–9^, enhancing cooling effectiveness and maximizing cooling capacity utilization while reducing ventilation energy consumption.

Localized jet ventilation within the deep and confined spaces of mines has garnered significant attention due to its potential to address thermal issues. Studies have revealed that the thermal sensation of the upper part of the human body (head and chest) has the most significant influence on overall thermal sensation^10–12^. Therefore, positioning jet ventilation in the area between the chest and the head of mine workers has shown positive cooling effects. Wang et al.^13^ utilized the relative warmth index (RWI) and percentage of dissatisfaction (PD) as evaluative metrics for dynamic thermal comfort, analyzing the characteristic thermal comfort dynamics beneath the air conditioning air supply nozzle under unsteady crossflow conditions. Furthermore, Xia et al.^14^ found that high turbulence intensity in air ventilation requires smaller air velocity for human body thermal comfort compared to low turbulence intensity, indicating that controlling the non-uniform thermal environment with high turbulence intensity can decrease human body discomfort in high-temperature thermal conditions^15–17^. Consequently, the implementation of localized jet ventilation in mines can help significantly improve the thermal comfort of mine workers, especially in the presence of piston wind, which increases the turbulence intensity in the mine’s thermal environment.

Researching localized jet ventilation technologies is critical in confined mine spaces to ensure safe mining production, given the prevalent high-temperature issues and low-speed crossflow circumstances. The jet in crossflow (JICF) phenomenon becomes involved when the incident occurs in the horizontal crossflow external environment, resulting in a complex and diverse flow field^18^. This JICF phenomenon finds diverse engineering applications, including engine nozzle cooling, air film cooling, fluid mixing, pollution discharge, environmental control, ventilation, and biomedical^19–26^. However, in the confined mine tunnel space, low-speed crossflow for oxygen and dust removal is always present, with the allowable crossflow speed of the coal mining face range being 0.25–4 m/s according to the Chinese Coal Mine Safety Regulations^27^. Consequently, the airflow organization of jet ventilation is significantly influenced by the low-speed crossflow in the external environment^28^, leading to complex changes in flow patterns within the confined mine spaces and the formation of typical jet ventilation in crossflow (JVIC) as shown in Fig. 1^29–31^. The jet is deflected by the thrust of the crossflow, and the entire jet can be divided into three sections: (I) Initial section: There is a potential flow core area near the jet exit. From the jet exit to the end of the potential flow core zone is the initial section of the jet, where the flow direction of the jet is basically along the inject direction, and the deflection is minimal; (II) Deflection section: From the end of the potential flow core area to the jet direction gradually deflects from orthogonal to the crossflow to parallel with the crossflow. (III) Run-through section: After the deflection section, the flow direction of the jet is basically consistent with the direction of the crossflow. The interactions of jet ventilation, crossflow, and confined mine space result in important flow phenomena such as entrainment, flow-around, mixing, and separation, leading to complicated changes in the flow-field structure, including the formation of horseshoe vortex, shear-layer vortices, counter-rotating vortex pair (CVP), and wake vortex^32^. These complicated vortex events cause significant changes in the flow-field structure in confined spaces, making the implementation of jet ventilation more challenging^18^. The effect of injecting into a turbulent boundary layer in a low-speed ratio crossflow was examined by Gopalan et al.^33^, who studied the flow structure and accompanying wall pressure fluctuations in detail. Likewise, Rajappan and Mahalakshmi^34^ investigated the effect of turbulence on a flat plate in an incompressible flow and determined that the shear layer between the jet and the main flow induced a strong intermittent flow structure. Furthermore, Tricouros et al.^35^ found that unstable rectangular jets into a flat laminar boundary layer formed stronger coherent flow vortices compared to steady jets. Williams et al.^36^ utilized a wall model large eddy simulation approach to compare the penetration and mixing capabilities of steady and sinusoidally pulsed jets, showing that sinusoidally pulsed jets are more conducive when momentum flux ratios are equivalent. Finally, Ho et al.^37^ used the Reynolds-averaged Navier–Stokes (RANS) equations to study the three-dimensional flow of a circular synthetic jet interacting with a turbulent crossflow, and discovered that the ejected jet is accompanied downstream by a reversed-flow region.

Flow visualization techniques have been extensively utilized for the examination of jet flow and diffusion characteristics, as evidenced by numerous studies^38–41^. Researchers commonly employ methods such as injecting smoke into the flow-field and utilizing laser or bright lights for flow visualization^42^. These visualization approaches have proven instrumental in the comprehensive analysis of jet flow and diffusion characteristics. Importantly, the references to flow-field visualization technology serve as valuable insights for guiding the experimental methodology in this study. Specifically, smoke flow visualization techniques were considered in the experimental design to observe flow features. For instance, smoke flow visualization techniques effectively reveal the flow morphology of a jet after its interaction with the crossflow^43^. Shoe et al.^44^ utilized laser-induced fluorescence (LIF) and smoke flow visualization imaging techniques to investigate the collision of two impinging jets, demonstrating the effectiveness and efficiency of such methods in exploring complex flows. Similarly, Kelso et al.^45^ elucidated the nature of jet structures using dye tracers in water channels and smoke-wire in wind tunnel experiments, providing valuable insights into the downstream development of CVP. Furthermore, Fric and Roshko^46^ employed laser sheet illumination of smoke to examine vortex structures in jet wakes, highlighting the connectivity between jet flow and wall boundary layer vortices. Carcasci et al.^47^ utilized various techniques, including smoke techniques, oil and dye techniques, and thermochromic liquid crystal techniques, for experimental flow visualization, offering insights into the flow pattern of subsonic impinging jet systems on a flat plate. Similarly, Khouygani et al.^48^ investigated the time-averaged flow regime and transient windward-side shear-layer vortex dynamics pattern of a backward-sloping elevated lateral jet using a laser-assisted smoke display technique in an open-loop wind tunnel. Additionally, Pastrana et al.^49^ conducted measurements using time-resolved particle image velocimetry (TR-PIV) to study the flow structure and flow characteristics of transverse impinging twin jets in confined laminar crossflow, providing valuable insights into the confinement effects of the confined jets.

However, the current research on local jet ventilation in mines does not take into account the influence of the crossflow on the airflow organization of jet ventilation, making controlling the localized jet ventilation difficult, and the improvement of the cooling effect and cooling efficiency is not significant. The study originates from the observed changes in flow structures due to crossflow influences on jet ventilation in confined mine spaces, similar to issues prevalent in scenarios such as subway tunnels, and the transition areas of factories, as well as other jet ventilation influenced by crossflow. Therefore, this paper aims to investigate the characteristics of velocity field distribution and time-averaged flow patterns of jets interacting with crossflows in confined spaces by wind tunnel experiments. The velocity field along the -z direction was measured for various jet exit diameters (*d*) and flow velocity ratios (*R*) to study the velocity distribution characteristics of the flow-field. Laser-assisted smoke flow visualization was used to examine the time-averaged flow characteristics of jets interacting with crossflows in confined spaces. Based on the experimental findings, the study discusses the flow-field distribution of JVIC and the mechanisms behind flow phenomena in order to provide significant theoretical insights for the design of local jet ventilation in confined spaces.

## Experimental setup

### Apparatus

**Figure 2:**
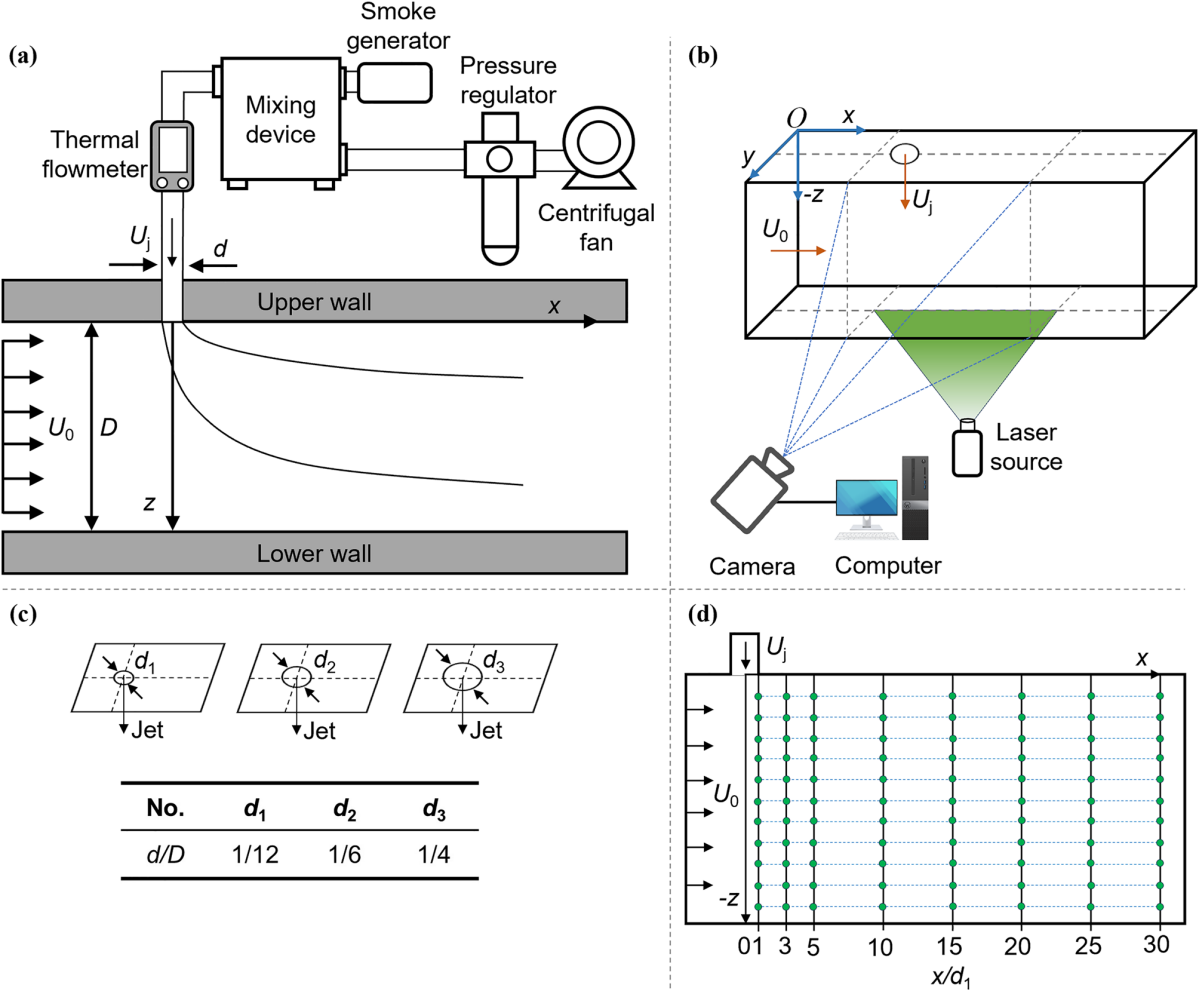
Schematic of the experimental setup: (**a**) test section setup; (**b**) flow visualizations setup; (**c**) jet exit diameters setup; (**d**) velocity measurement point setup.

The schematic configuration of the experimental setup is shown in Fig. 2. The wind tunnel used for the experiments was an open-loop suction-type wind tunnel, containing a test section with dimensions of 1500 mm (length) × 120 mm (width) × 120 mm (height), constructed from transparent acrylic panels to enable visualization of the smoke flow. This specific test section of the rectangular wind tunnel met the requirements for the diffusion of the deflected jet. To minimize the influence of the wall boundary layer on the flow, the central point of the jet exit was positioned along the central axis of the test section. Additionally, the circular jet orifice, with equivalent diameters *d* = 10 mm, 20 mm, and 30 mm, was located on the upper wall of the test section, perpendicular to the horizontal crossflow direction, and directed into the crossflow at a 90° angle to the plane of the test section's lower wall, as depicted in Fig. 2c. These coordinates were defined with the origin at the center of the jet exit in a Cartesian coordinate system, with the transverse direction (crossflow direction), corresponding to the *x*-coordinate, the lateral direction to the *y*-coordinate, and the axial direction (jet direction) to the *z*-coordinate. For the purpose of rendering all coordinates dimensionless, the *d*_1_ = 10 mm was utilized for this study. The ratio of the test section's height in the *z* direction to *d*_1_ was *z/d*_1_ = 12. Measurement holes for flow velocity were then established on the lower wall at *x/d*_1_ = 1, 3, 5, 10, 15, 20, 25, and 30, as shown in Fig. 2d. Utilizing anemometers, time-averaged flow velocity in the axial direction was measured at 11 measurement points established along the axial direction at the 1*d*_1_ interval. During the experiments, the anemometer was inserted into the monitoring orifices on the lower wall of the test section to measure flow velocity and was then retracted to the lower base plate after each measurement to prevent interference with the flow field. Pre-experimentation was conducted to exclude the impact of the boundary layer on the flow at the wall of the wind tunnel test section. Moreover, pre-experiments were undertaken to investigate the flow pattern following the interaction between the crossflow and the jet for *R* and *d/D*, demonstrating no impact from the boundary layer on the wind tunnel wall.

### Flow visualization

In order to visualize the flow as shown in Fig. 2a, a smoke mixing device constructed in-house was installed in the ductwork connecting the fan to the flowmeter. This device effectively blends smoke with air, thereby improving the visualization of the flow pattern. The smoke mixing device, connected to a 1500 W smoke generator (1500 FOGGER), the smoke mixing device produces homogeneous smoke with excellent followability and resolution, making it easily visible under the green laser. During the experiment, the smoke from the smoke generator was uniformly mixed with the airflow from a variable frequency centrifugal fan before being released from the jet exit.

Figure 2b displays the setup with a stationary laser was positioned bottom the test section, emitting a continuous laser sheet intersecting the central plane of the jet. The laser scatters the smoke flow, thereby enabling the visualization of the airflow pattern resulting from the interaction of the crossflow and the jet. A 532 nm wavelength green laser (13GCN3) was selected as the light source for flow visualization. This laser emits a horizontal green light measuring 13 mm × 62 mm and has a maximum output power of 400 mW. The width of the green laser sheet, adjustable in the range of 1–10 mm, was set at 1 mm for this study. By using optics, The laser beam was expanded into a planar laser sheet with a thickness of about 0.5 mm in the *x*–*z* plane, aligned vertically with the centerline of the *x*–*y* plane of the upper wall of the test section to facilitate side observation of the smoke flow pattern. To ensure clear visualization of the smoke flow pattern, this study employed a high-speed imaging technique to rapidly and repeatedly capture the smoke flow pattern.

A Canon EOS 800D digital camera was utilized to capture time-averaged (long-exposure) flow images by extending the exposure time. Equipped with a built-in CMOS sensor featuring a maximum resolution of 6000 × 4000 pixels, the camera provided a spatial resolution of 0.085 mm/pixel for the time-averaged flow images. A 6 s long-exposure time was selected to accurately capture the smoke flow pattern. The repetition of smoke visualization experiments and the capture of time-averaged flow images were carried out to verify the consistency of the collected flow patterns and to ensure the reliability and accuracy of the experimental results.

### Velocity measurement

The jet's exit velocity *U*_j_ was determined by a flow generated by a variable frequency centrifugal fan through an ASAIR thermal gas flowmeter (AFM07) with an accuracy of ± 3% F.S. The flow velocity distribution within the test section was measured by a hot-wire anemometer (TSI-9535), which anemometer had a reading accuracy of ± 3% (± 0.015 m/s) and a resolution of 0.01 m/s. Each measurement point was tested for 2 min with a time interval of 1 s between measurements, resulting in 120 sets of flow-field velocity. The measured flow rate of the thermal gas flowmeter was then used to calculate the jet's exit velocity *U*_j_ as the ratio of the flow velocity to the exit cross-sectional area of the jet. Subsequently, in this study, the time-averaged value of the measured velocity field was utilized to represent the velocity magnitude of the jet post-interaction with the crossflow. The time-averaged flow velocity $\overline{u}$ was presented in Eq. (1).

1 $$\overline{u} = \frac{1}{N}\sum\limits_{i = 1}^{N} {u_{i} } .$$

### Test conditions

This study aims to investigate the flow characteristics of JVIC in confined mine spaces through flow visualization experiments. Velocity gradient fields were measured for differing *R* and *d/D* values to analyze the flow-field distribution of JVIC. The degree of bending of JICF was found to be primarily associated with the initial momentum ratio, and in the case of a pure jet, it was influenced by the parameter *R*. In this experiment, both the crossflow and the jet consisted of air with the same density, establishing the experiment as a pure jet experiment where the kinematic viscosity *v*_j_ = *v*_o_. The Reynolds number (*Re*) of the jet (*Re*_j_ = *U*_j_*d*/*v*_j_) and the crossflow (*Re*_0_ = *U*_0_*D*/*v*_0_) were defined, with the ratio *R* = *U*_j_*/U*_0_ representing the flow velocity of the jet to the crossflow, where *U*_j_ represents the average flow velocity at the jet exit and *U*_0_ denotes the average flow velocity at the crossflow entrance. Under steady-state conditions, the entrance Reynolds number was calculated based on the equivalent diameter of the jet exit and average flow velocity. The influence of buoyancy was disregarded due to the crossflow velocity exceeding 0.1 m/s^50^. The experimental conditions included a crossflow velocity of *U*_0_ = 2 m/s, a Reynolds number of *Re*_0_ = 15,226, and a turbulence intensity of 0.5% in the crossflow. Three different jet exit diameters (*d* = 10 mm, 20 mm, and 30 mm) were used for the experiments, resulting in non-dimensional jet exit diameters (*d/D*) of 1/12, 1/6, and 1/4, respectively. The experiments were also conducted under normal temperature and pressure, and detailed experimental conditions can be found in Table 1.

**Table 1:** Experimental conditions.

*d/D*	*U*_j_ (m/s)	*R*	*Re*_j_
1/12	2	1	1269
	4	2	2538
	6	3	3806
	8	4	5075
1/6	2	1	2538
	4	2	5075
	6	3	7613
	8	4	10,151
1/4	2	1	3806
	4	2	7613
	6	3	11,419
	8	4	15,226

## Results and discussion

### Time-averaged flow characteristics

**Figure 3:**
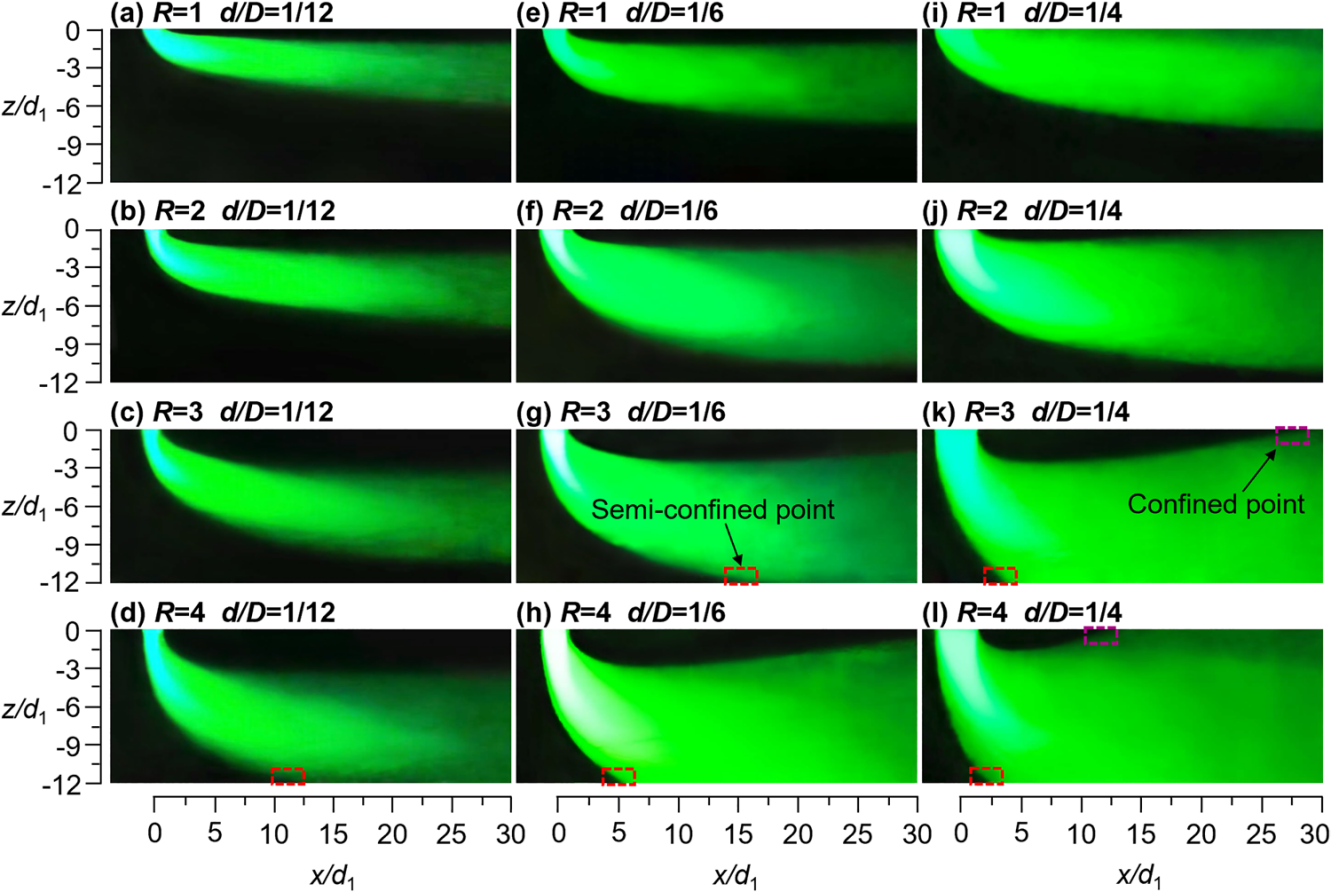
Time-averaged flow images with an exposure time of 6 s.

Figure 3 shows the time-averaged smoke flow distribution characteristics in the *x*–*z* plane, flowing along the crossflow direction. In confined mine spaces, under the influence of crossflow, vertical incidence jet ventilation deflects the flow trajectory from the -*z* direction to the *x* direction (crossflow direction). As the jet deflects, the diffusion of JVIC in the *x* direction gradually transitions to the *z* direction, as does its penetration. The flow trajectory of jet ventilation forms an “air lake” phenomenon, with the area covered by the air lake being determined by the diffusion capacity of JVIC. This highlights the need to study the influence of crossflow on airflow organization in confined mine spaces. Notably, the deflection angle (*θ*_j_) is defined as the angle between the tangent direction of outer boundary of the jet and the direction of incidence. It is observed that *θ*_j_ decreases continuously as *R* or *d/D* increases, indicating a negative correlation between *R* or *d/D* and *θ*_j_. This phenomenon is attributed to the increase in *R* and *d/D*, which strengthens the initial momentum of the jet incidence, thereby weakening the effects of the crossflow forces. Furthermore, as *θ*_j_ decreases, the JVIC moves toward the lower wall, leading to an increase in the mixing effect between the jet ventilation and the crossflow air, and an expansion of the area covered by the air lake. Therefore, *R* and *d/D* are key parameters in controlling the flow pattern and air lake of JVIC.

The smoke vortex (Coanda Effect) formed by the JVIC deflection attached to the upper wall vanishes as shown in Fig. 3i, due to the overlapping during a long-exposure time (6 s). When *R* = 1 and *d/D* = 4, the increased incident smoke enhances the diffusion ability of the JVIC, causing it to attach to the upper wall, forming a wall-attached jet with a deflection section and a wall-attached section. However, the wall-attached air lake formed in this scenario only diffuses in the -*z* direction. With increasing *R*, the flow trajectory of JVIC gradually moves in the -*z* direction, and the inner boundary of JVIC is separated from the upper wall, forming a typical deflection jet with an initial section, a deflection section, and a run-through section. This forms a suspended air lake that can diffuse in the -*z* and *z* direction. When the initial momentum of JVIC increases to a certain extent, the outer boundary of the jet intersects with the lower wall to form a semi-confined point (SP) as shown in Fig. 3g. In the far-field region after the SP, JVIC attached to the lower wall, the deflected jet's run-through section transformed into a wall-attached section. Consequently, the flow pattern of the JVIC evolves from a deflected jet to a semi-confined deflected jet in the presence of SP. This signifies the loss of the deflection jet’s ability to deflect and diffuse in the -z direction due to the wall confinement effect. This semi-confined deflection jet forms a wall-attached air lake in the lower wall, which will eventually be completely transformed into mixing ventilation. Furthermore, When *R* = 4, as *d/D* increases, the SP continues to move in the -*x* direction and is located at *x/d*_1_ = 10, 5, and 2.5, respectively. This causes the deflection section in the *x* direction to continuously shorten and leads to an increase in the air lake mixing effect with the crossflow. The initial section of JVIC always lengthens during the transition of the flow pattern from wall-attached jet to semi-confined deflected jet, whereas the deflection section tends to lengthen first and subsequently shorten. The SP is linked to the maximum length of the JVIC deflection section. Notably, the diffusion capacity of the JVIC toward the -*z* direction is shifted to the *z* direction when the SP appears. As the initial momentum of the jet continues to increase, the inner boundary of the jet rapidly moves up the upper wall. Figure 3k shows that the inner boundary of the jet intersects the upper wall to form a constrained point (CP). Figure 3l shows that the far-field region after the CP is completely covered by the smoke flow, this means the diffusion capability of the jet is completely confined. Furthermore, no re-circulation flow structure was observed at the impingement site, where the jet traveled in the -*x* direction and interacted with the crossflow. In the JVIC, there are four flow modes under different *R* and *d*/*D* conditions, as shown in Fig. 3: attached-wall jet, deflected jet, semi-confined deflected jet, and confined jet. Figure 1 shows that the effective region for cooling underground mine workers is the head-neck. This local cooling mode, as opposed to full air cooling, enhances the cooling effect and the utilization efficiency of cooling capacity. The wall-attached and deflected jets generated by the JVIC create a stable and long-distance air lake, resembling the airflow organization of displacement ventilation. This airflow organization enables effective local cooling of the head-neck. When the SP is formed at the lower wall, the jet forms a wall-attached section requiring a higher initial momentum to cater to the localized cooling demand for the head-neck. Therefore, the effectiveness of the flow pattern after the semi-confined deflected jet for local mine ventilation needs to be further analyzed in comparison with the full-air ventilation mode. In summary, the air lake formed by JVIC provides a feasible design idea for localized jet ventilation in mines.

### Characteristics of flow-field velocity distribution

**Figure 4:**
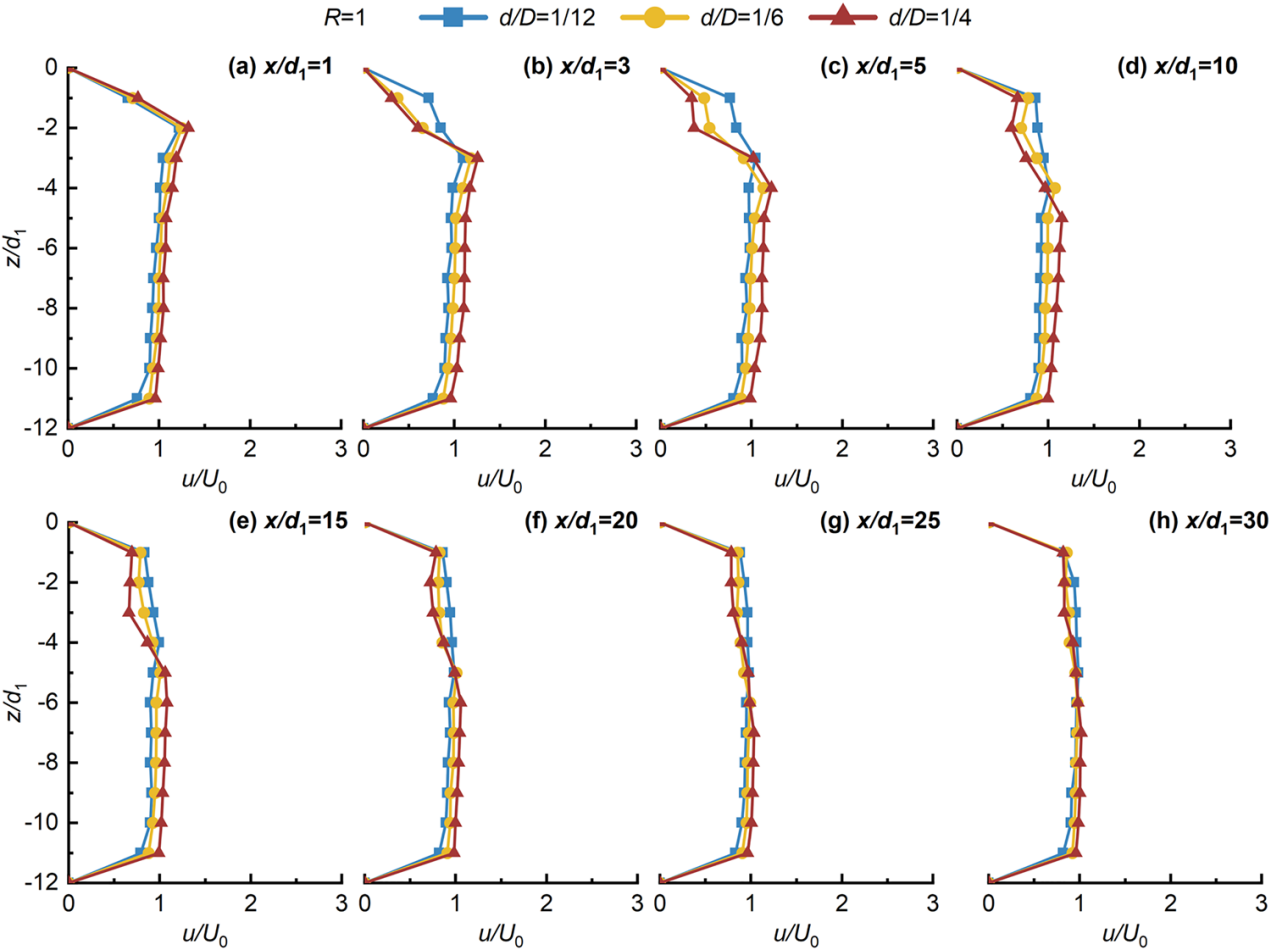
Variation of *u/U*_0_ in the *z* direction with *x/d*_1_ in the *x* direction for *d/D* = 1/12, 1/6, and 1/4 at *R* = 1.

**Figure 5:**
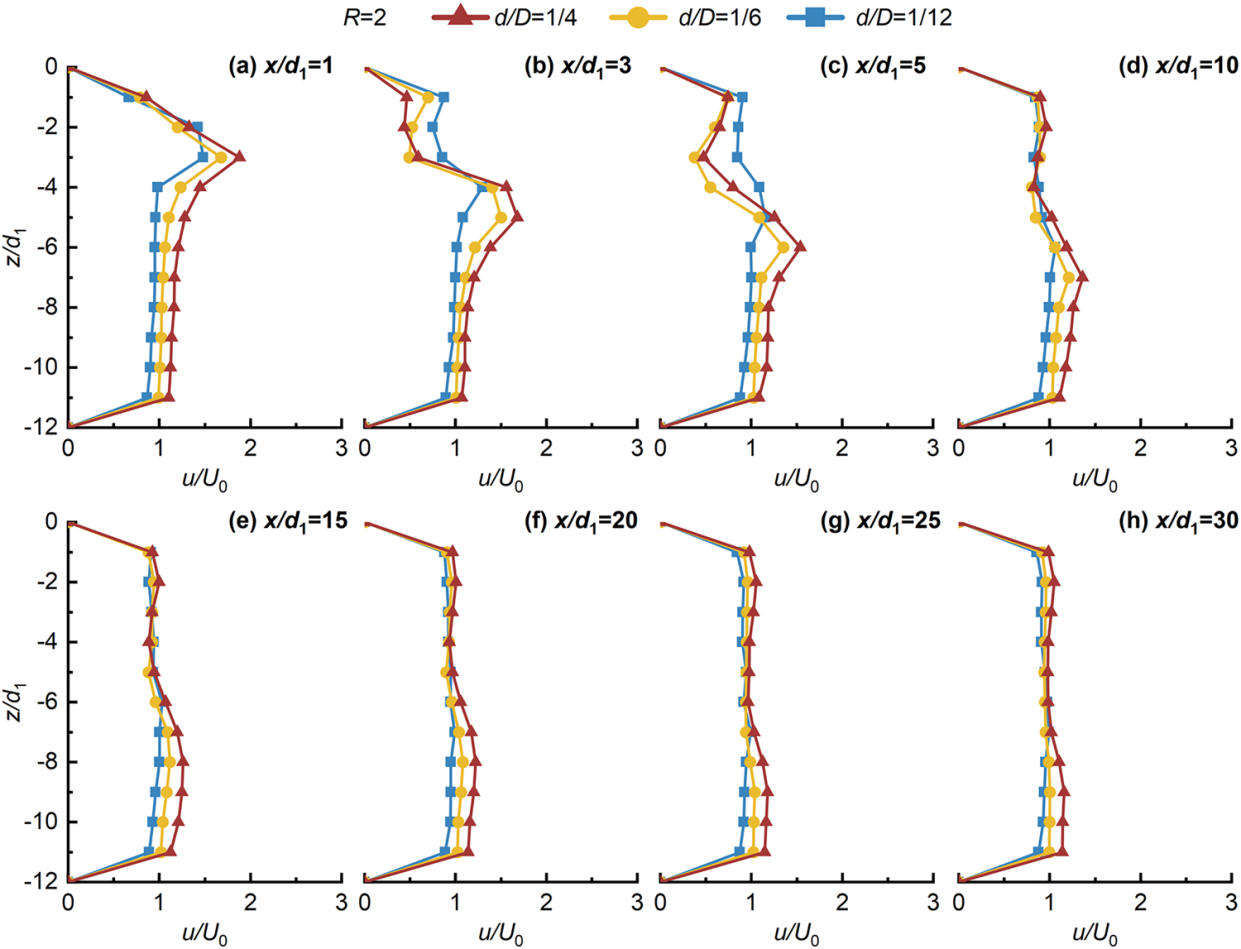
Variation of *u/U*_0_ in the *z* direction with *x/d*_1_ in the *x* direction for *d/D* = 1/12, 1/6, and 1/4 at *R* = 2.

**Figure 6:**
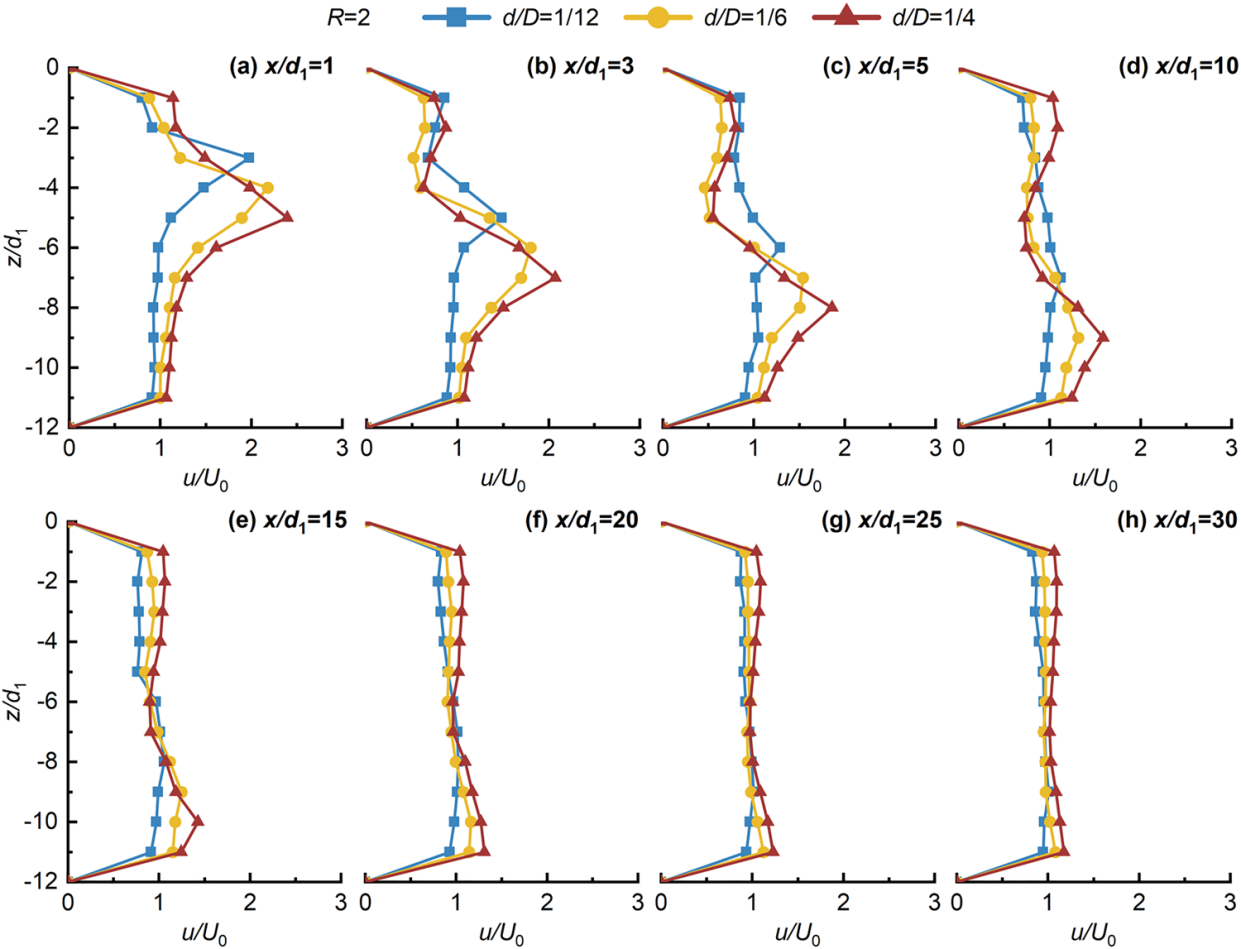
Variation of *u/U*_0_ in the *z* direction with *x/d*_1_ in the *x* direction for *d/D* = 1/12, 1/6, and 1/4 at *R* = 3.

**Figure 7:**
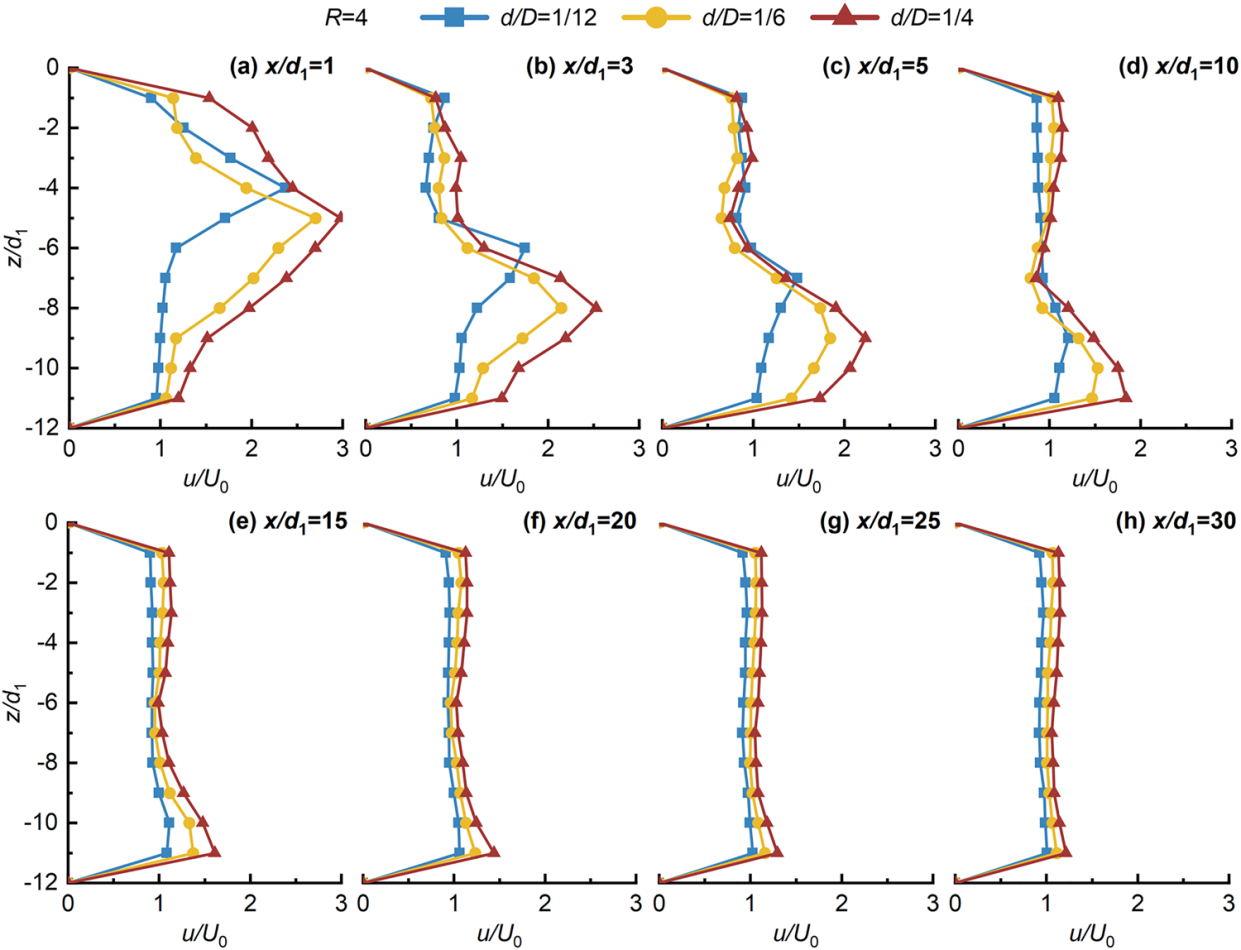
Variation of *u/U*_0_ in the *z* direction with *x/d*_1_ in the *x* direction for *d/D* = 1/12, 1/6, and 1/4 at *R* = 1.

Figures 4, 5, 6, 7 shows the non-dimensional flow velocity (*u/U*_0_) distribution in the *x* direction. Notably, the -*z* direction exhibits a pattern of decreasing, increasing, and then decreasing to *U*_0_ again. Furthermore, as *x/d*_1_ increases, the point of maximum non-dimensional flow velocity (*u/U*_0max_) shifts towards the -*z* direction. The hindering effects of the jet with crossflow lead to a significant flow-around phenomenon on both sides of the jet in the near-field region. However, in the far-field region, the distance between the point of *u/U*_0max_ and the lower wall does not significantly increase, indicating the gradual disappearance of the flow-around effect of the crossflow, and then the flow enters the uniform flow region. This suggests that the impact of the crossflow on the jet ventilation is mainly concentrated in the deflection section. Beyond the point of *u/U*_0max_ in the -*z* direction, the *u/U*_0_ distribution stabilizes and tends towards 1, signifying strong mixing effects of JVIC. Moreover, the secondary flow phenomenon steadily weakens with increasing *x/d*_1_. It is noteworthy that increasing *d/D* and *R* enhances the secondary flow phenomenon near the upper wall due to the increased initial momentum of the jet, amplifying the hindering effect of the jet. Consequently, the pressure difference on both sides of the jet results in a more significant flow-around phenomenon in the deflected section. Simultaneously, the wall confinement effect intensifies both the pressure and vortex effects in the confined space, strengthening the secondary flow phenomena. As *R* and *d/D* increase, the point of *u/U*_0max_ = 1 gradually shifts towards the *x* direction, indicating a positive correlation between the jet’s *x* direction penetration ability and both *R* and *d/D*. This implies that increasing the initial momentum of the jet facilitates the air lake to cross a further distance in the *x* direction. As shown in Fig. 7, as *x/d*_1_ = 20 to 30 increases, the point of *u/U*_0max_ located at *z/d*_1_ = 11 due to the jet's inability to continue deflecting in the -*z* direction under the impact of the wall confinement effect. This infers the formation of the SP on the lower due to the wall confinement effect wall, and then the development of a wall-attached air lake. Notably, as *R* and *d/D* increase, the SP will move along the -*x* direction, further shortening the jet deflection section, and enhancing the penetration capability of JVIC in the *x* direction increases. Consequently, increasing *R* and *d/D* expands the obstruction range to the crossflow by jet ventilation. This underscores that the initial momentum of the jet directly influences the magnitude of *θ*_j_ in the *z* direction. When the initial momentum of the jet is high, the crossflow's pushing effect on the jet ventilation is not significant, resulting in a large obstacle pressure difference between the jet and the crossflow, and a significant mixing between the two fluids. Furthermore, the impedance of the jet on the crossflow decreases in the *x* direction due to the dissipation of jet ventilation energy, leading to intensive mixing between the two fluids forming a uniform and steady flow of air lake. Consequently, the decay in the axial velocity of the jet occurs at a farther distance, illustrating that the wall confinement effect in the confined space delays the rate of *u/U*_0max_ decay.

### Characteristics of jet axis trajectory in confined space

The initial momentum of the jet ventilation has a major impact on its *θ*_j_, while in the case of a pure jet, the degree of deflecting was influenced by *R*. This study defines the jet axial trajectory as the line connecting the points of *u/U*_0max_ along the *x* direction to depict the trend effectively. The jet axial trajectory formulae (2) in this research are mainly based on the equation proposed by Wu^51^.

2 $$\left( {z{/}d_{1} } \right) = CR^{a} \left( {x{/}d_{1} } \right)^{b} .$$

**Figure 8:**
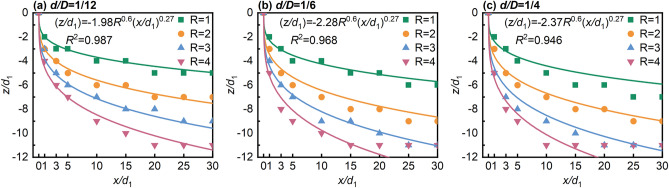
Variation of jet axis trajectories with *x/d*_1_.

Figure 8 shows the empirical formulae for the jet axial trajectory, derived in this study by incorporating Eq. (2) and the range of constant values from Table 2. The fitted function was validated based on the criterion of goodness-of-fit (adjusted R-squared) greater than 0.8, with R-squared values of 0.987, 0.966, and 0.93, respectively, indicating an accurate depiction of the actual curve^57^. However, the adjusted R-squared decreases continuously with increasing *R* and *d/D* in the fitting results. Additionally, as the initial momentum increases, the point of *u/U*_0max_ constantly moves towards the lower wall, leading to located at *z/d*_1_ = 11 gradually increasing. This causes the lower wall to force the JVIC to deflect in the *x* direction, resulting in a wall-attached air lake, and shortening of the deflection section, transiting rapidly the deflection jet section into the wall-attached section. Consequently, the jet axial trajectory becomes linear after the SP. Furthermore, the jet axial trajectory shifts gradually to the -*z* direction as *R* and *d/D* increase, indicating that the obstruction effect produced by the jet ventilation is directly determined by the magnitude of *R* and *d/D*.

**Table 2:** Reference range of values of empirical formula constants for jet trajectories.

Constants/references	Wu et al.^51^	Lin et al.^52^	Wu et al. ^53^	Ahn et al.^54^	Yoon et al	Wang et al.^55^	Jiang et al.^56^
*C*	1.37	2.42	4.3	1.297	2.241	1.09	1.17
*a*	0.5	0.48	0.33	0.491	0.402	0.625	0.67
*b*	0.5	0.24	0.33	0.509	0.41	0.35	0.38

### Characterization of the jet axial velocity variation in confined space

**Figure 9:**
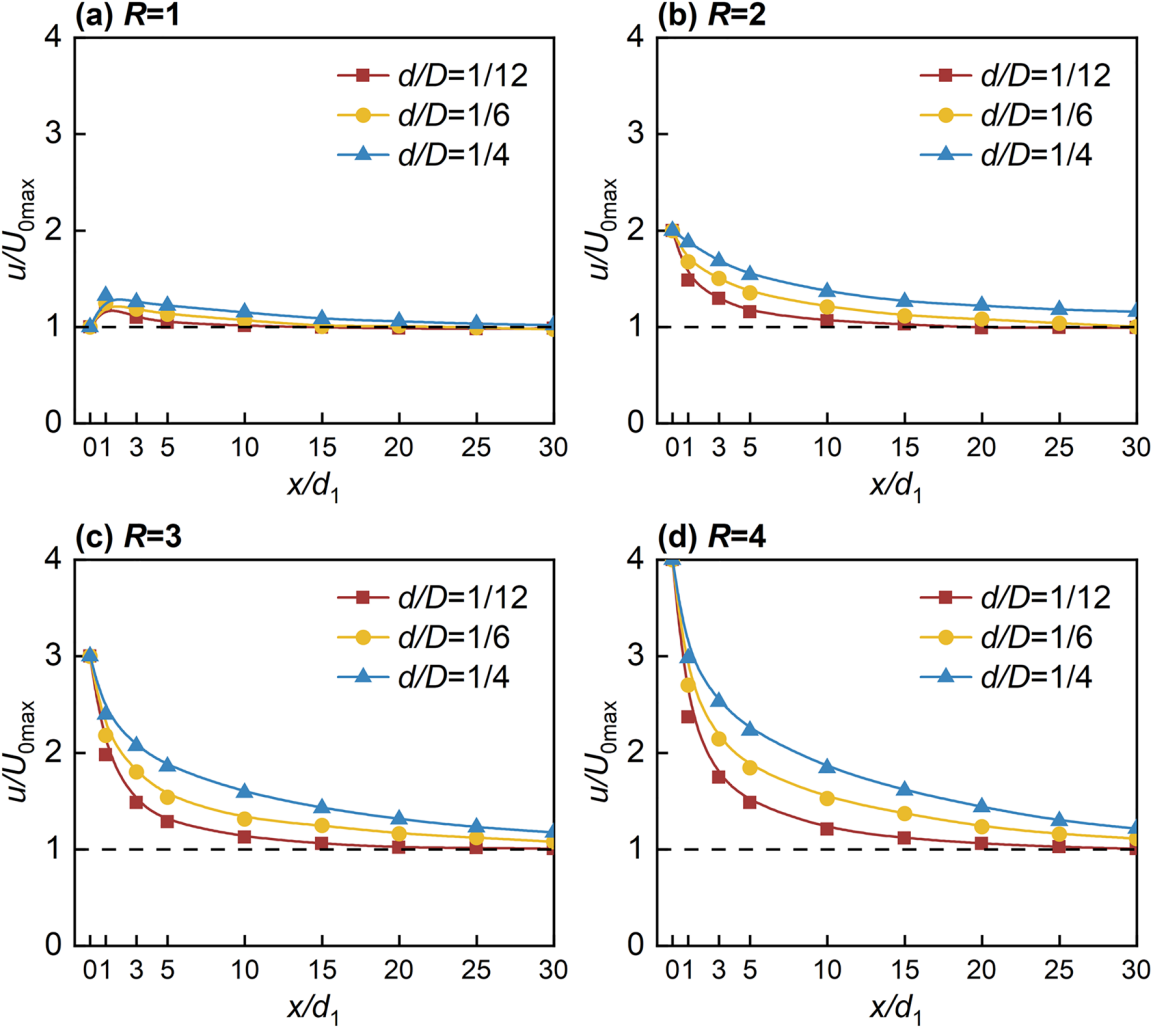
Variation of *u/U*_0max_ with *d/D* at different *R*.

Figure 9 shows the *x* direction distribution of the non-dimensional jet axial flow velocity (*u/U*_0max_). Figure 9a shows that the *u/U*_0max_ at *R* = 1 and *x/d*_1_ = 1 were 1.225, 1.27, and 1.32, respectively, all of which were significantly higher than the initial jet exit flow velocity. This increase can be attributed to the impact of the crossflow, which causes the jet to deflect despite having a lower initial momentum, thereby leading to an increase in *θ*_j_. Consequently, Significant secondary flow phenomena are observed due to strong entrainment and mixing between the jet and the crossflow near the jet's exit, resulting in a velocity superposition. On the other hand, Fig. 9b–d shows a rapid decaying trend in *u/U*_0max_, with the values gradually converging to 1 due to the constant mixing between the jet and the crossflow. The rapid decay is observed predominantly in the near-field region, whereas the values tend to level off in the far-field region. This phenomenon is attributed to the extensive hindering effect exerted by the jet in the initial and deflection sections on the crossflow, leading to strong entrainment and mixing between the jet and the hindered crossflow. Additionally, the momentum dissipation to counteract the pushing impact of the crossflow during the jet's deflection from the -*z* to the *x* direction is significant. Consequently, the flow-around in the far-field region gradually disappears, and the JVIC forms a more uniform flowing air lake, with mixing occurring on both sides of the air lake. The phenomenon of *u* approaching *U*_0_ is observed successively as *x/d*_1_ increases, with the positions indicated in Fig. 9a as *x/d*_1_ = 10, 20, and 30. However, for *R* = 2 and *d/D* = 1/4, Fig. 8b shows that at *x/d*_1_ = 30, the *u/U*_0max_ value remains at 1.16, indicating ongoing mixing between the air lake and the crossflow. As shown in Fig. 9c,d, it is evident that the distance covered by the air lakes in the *x* direction depends on the magnitude of the initial momentum, as the position where *u* approaching *U*_0_ moves farther away with increasing *R* and *d/D*. This implies that the distance covered by the air lakes in the *x* direction is contingent on the magnitude of the initial momentum.

### Characteristics of the distribution of the jet's inner and outer boundaries and its width in confined space

**Figure 10:**
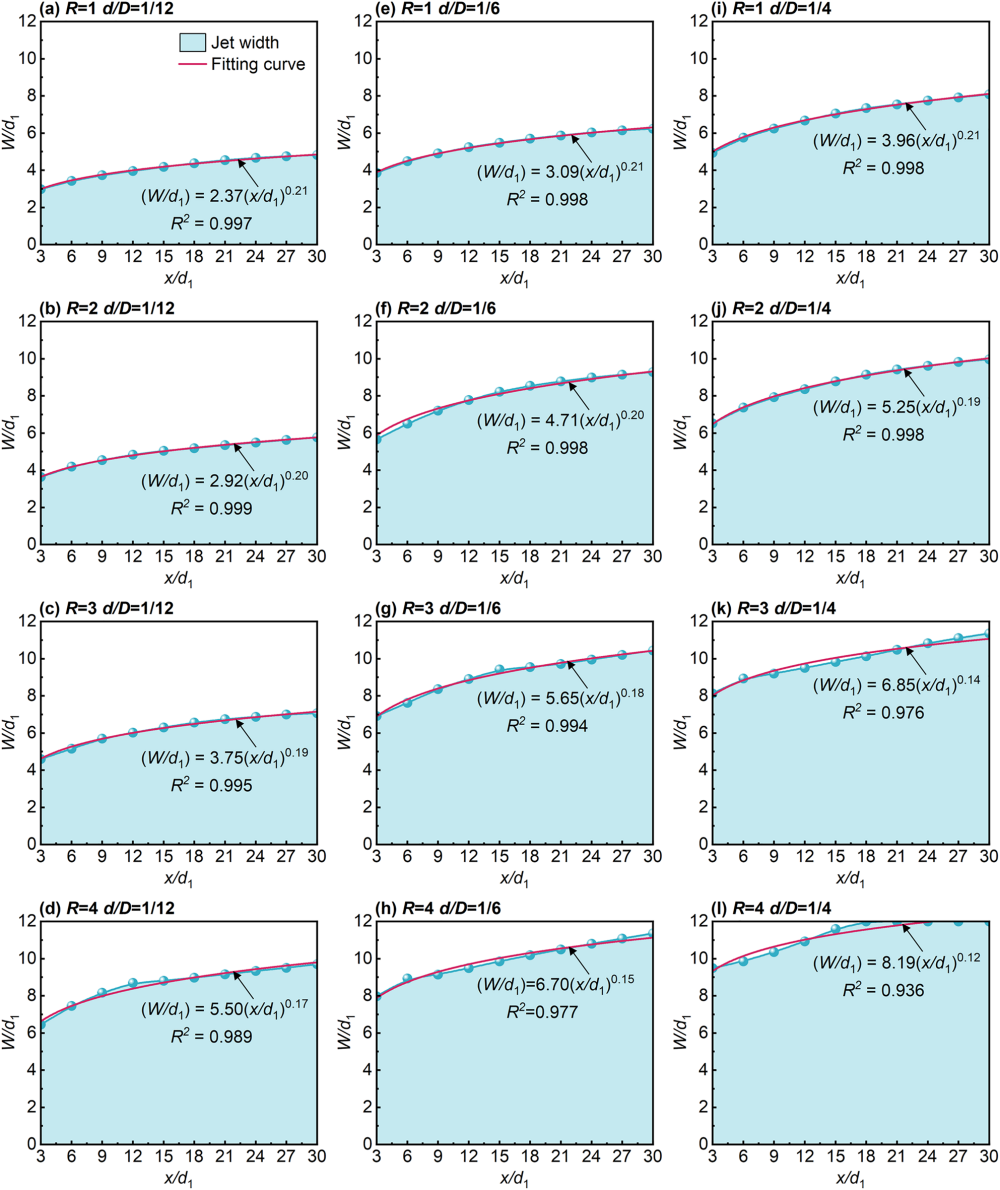
Variation of *W/d*_1_ with *d/D* at different *R*.

The area covered by the air lake in the mine is directly determined by the diffusion capability of JVIC. In this study, the jet width (*W*) was defined, representing the axial diffusion index of the JVIC, and the jet trajectory extraction method by Lan et al.^58^ was employed to obtain *W*. The methodology involved maintaining environmental and camera parameters consistent with actual conditions. Subsequently, grayscale processing and histogram equalization were applied to the time-averaged flow images to enhance clarity and contrast. The processed images underwent threshold segmentation processing, and edge detection techniques were used to extract the inner and outer boundary trajectory. Finally, the non-dimensional jet width (*W/d*_1_) of JVIC was determined by subtracting the corresponding non-dimensional coordinate (*z/d*_1_) of the inner and outer boundaries. Given that *W/d*_1_ was solely a function of *x/d*_1_ and independent of *R*^59^. As shown in Fig. 10, The empirical formulae (3) for *W/d*_1_ established applicable to the study were primarily based on those proposed by Subramanya and Ben Meftah^20,59^.

3 $$\left( {W{/}d_{1} } \right) = A\left( {x{/}d_{1} } \right)^{B} .$$

Figure 10 depicts the distribution of non-dimensional jet width with *x/d*_1_. it was observed that the adjusted R-squared of the empirical formulae for various *R* and *d/D* conditions was above 0.8, indicating conformity to formulae (3). Notably, as the jet forms an air lake and is attached to the lower wall, the adjusted R-squared begins to show significant decreases, and the SP moves in the -*x* direction as* R* and *d/D* increase. The inner boundary continuously moves toward the upper wall until the fully confined JVIC is formed on the lower wall. This confinement effect leads to a rapid diffusion in the *z* direction, enhancing the diffusion capability. The maximum value of the non-dimensional jet width (*W/d*_1max_ = 12) is reached when the inner boundary intersects with the upper wall to form CP. Furthermore, the *W/d*_1_ increases as *d/D* and *R* increase. Subsequently, the growth rate of *W/d*_1_ decreases due to the weakening of the mixing between the jet and the crossflow during the flow mode from the deflection section to the run-through section. This indicates a positive correlation between the axial diffusion capacity of JVIC and the jet's initial momentum, while the height of the confined space determines the upper limit of axial diffusion.

## Conclusion

The study investigates the time-averaged flow pattern and flow-field distribution characteristics of JVIC in confined mine spaces utilizing a laser-assisted smoke flow visualization technique and flow velocity measurements. Based on the results obtained, several conclusions can be drawn.

1. Firstly, it is observed that jet ventilation in confined mine spaces is subject to strong crossflow effects, leading to changes in the flow pattern. The influence of the crossflow results in the deflection of the jet. The air lake formed by JVIC in a confined space resembles the airflow organization of displacement ventilation, categorized as wall-attached air lakes (attached to upper or lower walls) and suspended air lakes. The air lake created by the wall-attached jet and deflected jet flow regimes can efficiently satisfy the head-neck local need for cooling control in mines. Furthermore, empirical formulae specific to this study were developed by fitting the jet axial trajectory and diffusion width. It is concluded that the key control parameters that influence the velocity distribution, flow trajectory, diffusion capacity, and penetration capacity of JVIC in confined mine spaces are the *R* and *d*/*D*.
2. Secondly, it is noted that the section of JVIC deflection first experiences growth and then rapidly shortens as the *R* and *d/D* increase. Significant secondary flow phenomena are observed in the JVIC deflection section, while the effect of flow mixing effect is minimal and uniform in the run-through or wall-attached sections. The initial momentum of the jet directly influences the deflection, diffusion, and penetrating capacity of JVIC. This is demonstrated by the deflected jet creating SP and CP with the lower and upper walls, respectively. The constraint impact of confined space gradually weakens the deflection and diffusion capacity of JVIC until it is completely lost. However, the confinement effect enhances the penetration ability of JVIC in the *x* direction, facilitating the flow of the air lake further away. Therefore, it is evident that the wall confinement effect significantly influences the flow characteristics of JVIC in confined mine spaces.

## Data Availability

The data that support the findings of this study are available from the corresponding author upon reasonable request.

## List of symbols

*D*: Equivalent diameter of test section (mm)
*d*: Equivalent diameter of jet exit (mm)
*x*: Position in transverse direction (crossflow direction)
*y*: Position in lateral direction of the test section
*z*: Position in axial direction (jet direction)
*N*: Total number of time sampling points (coefficient in Eq. (1))
*u*: Instantaneous velocity at measurement points (m/s)
*U*_0_: Crossflow velocity (m/s)
*U*_j_: Jet exit velocity (m/s)
*R*: Jet-to-crossflow velocity ratio (= *U*_j_/*U*_0_)
*Re*_0_: Reynolds number of crossflow (*Re*_0_ = *U*_0_*D*/*v*_0_)
*Re*_j_: Reynolds number of jet (*Re*_j_ = *U*_j_*d*/*v*_j_)
*v*_0_: Kinematic viscosity of crossflow
*v*_j_: Kinematic viscosity of jet
*I*: Turbulence intensity
*θ*_j_: Deflection angle of the jet
*W*: Jet width in axial direction
*C*: Coefficient for term of *R*^a^ in Eq. (2)
*a*: Exponent for *R* in Eq. (2)
*b*: Exponent for *x*/*d*_1_ in Eq. (2)
*A*: Coefficient for term of (*x*/*d*_1_)^B^ in Eq. (3)
*B*: Exponent for *x*/*d*_1_ in Eq. (3)
